# Machine learning-based analysis and prediction of meteorological factors and urban heatstroke diseases

**DOI:** 10.3389/fpubh.2024.1420608

**Published:** 2024-07-22

**Authors:** Hui Xu, Shufang Guo, Xiaojun Shi, Yanzhen Wu, Junyi Pan, Han Gao, Yan Tang, Aiqing Han

**Affiliations:** ^1^School of Management, Beijing University of Chinese Medicine, Beijing, China; ^2^School of Humanities, Beijing University of Chinese Medicine, Beijing, China

**Keywords:** heatstroke, meteorological factor, machine learning, time series, DLNM

## Abstract

**Introduction:**

Heatstroke is a serious clinical condition caused by exposure to high temperature and high humidity environment, which leads to a rapid increase of the core temperature of the body to more than 40°C, accompanied by skin burning, consciousness disorders and other organ system damage. This study aims to analyze the effect of meteorological factors on the incidence of heatstroke using machine learning, and to construct a heatstroke forecasting model to provide reference for heatstroke prevention.

**Methods:**

The data of heatstroke incidence and meteorological factors in a city in South China from May to September 2014–2019 were analyzed in this study. The lagged effect of meteorological factors on heatstroke incidence was analyzed based on the distributed lag non-linear model, and the prediction model was constructed by using regression decision tree, random forest, gradient boosting trees, linear SVRs, LSTMs, and ARIMA algorithm.

**Results:**

The cumulative lagged effect found that heat index, dew-point temperature, daily maximum temperature and relative humidity had the greatest influence on heatstroke. When the heat index, dew-point temperature, and daily maximum temperature exceeded certain thresholds, the risk of heatstroke was significantly increased on the same day and within the following 5 days. The lagged effect of relative humidity on the occurrence of heatstroke was different with the change of relative humidity, and both excessively high and low environmental humidity levels exhibited a longer lagged effect on the occurrence of heatstroke. With regard to the prediction model, random forest model had the best performance of 5.28 on RMSE and dropped to 3.77 after being adjusted.

**Discussion:**

The incidence of heatstroke in this city is significantly correlated with heat index, heatwave, dew-point temperature, air temperature and *zhongfu*, among which the heat index and dew-point temperature have a significant lagged effect on heatstroke incidence. Relevant departments need to closely monitor the data of the correlated factors, and adopt heat prevention measures before the temperature peaks, calling on citizens to reduce outdoor activities.

## 1 Introduction

Heatstroke is a series of clinical symptoms caused by fluid and electrolyte disorder, acid-base imbalance, and dysfunction of the thermoregulatory center and the cardiac and cerebral nerves due to prolonged body exposure to high temperature and heat radiation (1). Heatstroke may occur when the temperature exceeds 36°C and the relative humidity exceeds 58% (2). With increasing greenhouse gas emissions and El Nino events, the probable appearance of a year with extreme heat within the next 5 years is as high as 98 per cent (3), which may lead to a significant rise in the number of heatstroke victims. Heatstroke predisposes the heart to added burden, triggering neurological organ damage and systemic inflammatory response syndromes, which can lead to a dramatic increase in the risk of death (4, 5). Compared with the 1986–2005 average, Chinese people experienced 7.85 more heatwave days on average in 2021 (6), and the number of deaths associated with high-temperature heatwaves in China has risen rapidly since 1979 (7). Therefore, the analysis of the impact of meteorological factors on the incidence of heatstroke is crucial for preventing heatstroke and maintaining public health.

Previous studies exploring the effect of meteorological factors on heatstroke have focused on key variables such as temperature and humidity, and analyzed them with a single statistical method. Kumar et al. used simple statistical estimation methods to analyse the effect of heat exposure on human health in the Indian region (8). Wang et al. (9) used a random-effects Poisson regression model to estimate the relative risk (RR) of hospital admission for heatstroke in heatwave weather vs. non-heatwave weather, and had found that the more severe and prolonged the heatwave, the higher the RR value. Li et al. (10) used a zero-inflated Poisson regression model with a logistic distribution to analyze the influence of daily maximum temperature on the occurrence of heatstroke, considering factors such as gender, age, and the severity of heatstroke. In recent years, machine learning algorithms have been gradually applied to the environmental and public health fields. Compared with traditional statistical methods, machine learning algorithms have stronger data processing and model generalization capabilities, and are able to better capture complex non-linear relationships and interactions between multiple factors. The application of machine learning algorithms has made significant progress in the study of the relationship between heatstroke and meteorological factors. Han et al. used correlation analysis and random forest model to analyze the relationship between meteorological variables and heatstroke search index in 333 Chinese cities from 2013 to 2020 (2). Wang et al. used a random forest model to predict heatstroke occurrence for heatwave based on 3 years' data in typical cities with high temperatures in China, which had better performance than the traditional linear regression model. The results indicates that meteorological factors play the most significant role in the model's estimation of the parameters evaluated (11). In addition, some studies have found that the high temperature and high humidity of *sanfu* (the dog days of summer in China) is closely related to the occurrence and treatment of many diseases, such as heatstroke and asthma. Zhu et al. (12) conducted a study on the treatment of asthma by acupuncture, and came to the conclusion that the treatment of the disease is related to *sanfu* in China. Although a number of studies have examined the relationship between heatstroke and meteorological factors, relatively few studies have combined multiple meteorological factors to analyse heatstroke disease in a multidimensional manner.

Therefore, a variety of characteristic data, such as meteorological factors, comprehensive indicators and time series of *sanfu*, were incorporated in this study to reveal the influencing factors of the onset of heatstroke more comprehensively. Adopting a variety of machine-learning algorithms, this study tried to fully exploited the potential information of the data, and has selected the optimal model for making predictions by comparing the performance and prediction effects of different algorithms, so as to improve the accuracy and reliability of the predictions. In addition, to understand the lagged effect and non-linear relationship of heatstroke incidence in a deeper way, this study used the traditional statistical method of Distributed Lag non-linear Model (DLNM) for analysis, thus describing more accurately the relationship and pattern between meteorological factors and heatstroke incidence, which provided an important basis for formulating effective early warning strategies and constructing prediction models. This study has a positive effect on reducing the incidence of heatstroke and protecting public health.

## 2 Materials and methods

### 2.1 Data source and variables

This study used data from *Data on heatstroke incidence and meteorological factors in a southern city from May to September in 2014–2019* created by the Chinese Center for Disease Control and Prevention (CDC). The dataset was collected and filled in through the existing monitoring system, integrating data from multiple testing sources, and was released after review by experts, thus reliable data quality. The data contains 919 records and 11 features.

In order that the characteristic data could be better used for the prediction of heatstroke, we calculated the data of daily average air temperature, daily maximum air temperature and relative humidity, and obtained two commonly used comprehensive meteorological indicators, namely heat index and dew-point temperature.

Heat index, i.e., apparent temperature, taking into account the combined effect of both air temperature and relative humidity, refers to the fact that at high temperatures, when the relative humidity is increased, the temperature felt by the human body is higher than the actual temperature. Research has shown that at the same temperature, different relative humidity levels will give individuals different levels of comfort, which in turn will have different impacts on human health (13, 14). The formula for its calculation is as follows:


$$\begin{array}{l} HI=c_{1}+c_{2}T+c_{3}\left[RH\right]+c_{4}T\left[RH\right]+c_{5}T^{2}+c_{6}{\left[RH\right]}^{2}+\\ \;c_{7}T^{2}\left[RH\right]+c_{8}T{\left[RH\right]}^{2}+c_{9}T^{2}{\left[RH\right]}^{2} \end{array}$$

Dew-point temperatu3ature at which the atmosphere is saturated with water vapor when it is cooled without changing its pressure or vapor content (15). When the dew-point temperature is low, the air temperature or the relative humidity will also be low, either of which can facilitate effective heat dissipation by the human body, thereby reducing the risk of heatstroke. This study employed the Magnus formula to calculate dew-point temperature, utilizing values of *a* = 17.27 and *b* = 237.7°C.


$$\begin{array}{r} T_{d}=\frac{b\gamma \left(T,RH\right)}{a-\gamma \left(T,RH\right)}\\ \gamma \left(T,RH\right)=\frac{aT}{b+T}+ln\left(RH/100\right) \end{array}$$

High-temperature heatwaves were included in our study as features as well. A high-temperature heatwave is a complex atmospheric phenomenon that usually refers to a series of consecutive hot days (16). According to the criteria of China Meteorological Administration (CMA), a daily maximum temperature ≥35°C is considered as a “high-temperature day,” and three or more consecutive high-temperature days are considered as a high-temperature heatwave. Based on this standard, the daily maximum temperature in the original data was converted, and those who were in a high-temperature heatwave were assigned a value of 1 and those who were not 0.

In addition, the effect of *sanfu* timing characteristics on the number of heatstroke victims was examined. According to the theory of TCM, *sanfu* refers to the three specific periods of the Chinese lunar year from July to August. Specifically, there are *toufu* (the beginning part of *sanfu*) and *mofu* (the ending part of *sanfu*), each lasting precisely 10 days, as well as *zhongfu* (the middle part of *sanfu*), which lasts either 10 or 20 days (17). *Sanfu* has typical climate characteristics such as high temperature, low air pressure, high humidity and low wind speed. The three variables, *toufu, zhongfu*, and *mofu*, were assigned 0 and 1 according to the dates of *sanfu* in each year. “0” means it is not in the corresponding period, while “1” means it is. The specific time of sanfu from 2014 to 2019 is shown in Supplementary Table 1.

The finalized dataset comprised primarily temporal variables such as the onset date, year, month, day, weekday, holiday status, and periods of the *sanfu*. It also encompassed meteorological variables including daily average temperature, daily maximum temperature, relative humidity, heat index, dew-point temperature, and high-temperature heatwaves. Additionally, it featured daily total counts of heatstroke incidents and the total population, amounting to a total of 17 variables. The individual variables and their descriptions are shown in Supplementary Table 2.

### 2.2 Method

Based on a number of meteorological characteristic data and time characteristic data, a distributed lag non-linear model was used to analyse the effects of meteorological factors, such as temperature, humidity and their integrated indicators, high-temperature heatwaves and the *sanfu* time series, on the incidence of heatstroke. The results of the analyses were combined to construct a heatstroke early warning model through machine learning models such as random forest, which provided a basis for preventing the occurrence of heatstroke.

#### 2.2.1 Distributed lag non-linear models

Previous study has shown lag in effect of heat on heatstroke (18), and that the relationship between heat and mortality in the population was mostly non-linear with a “J” curve (19). Therefore, in this paper, a distributed lag nonlinear model (DLNM) was used to fit the relationship between the number of heatstroke occurrences and meteorological factors. The DLNM describes the distribution of the dependent variables in the independent and lagged dimensions by constructing a cross-base, and is now mostly used in analyses of the effects of meteorological factors (20, 21). The formula for its calculation is as follows:


$$\begin{array}{l} \log E\left[Y_{t}\right]=\alpha +cb\left(x_{i},lag\right)+ns\left(date,10^{*}1\right)+dow+holiday \end{array}$$

*E*[*Y*_*t*_] was the number of daily heatstroke occurrences on day *t*, α was the intercept, cb(x_i_, lag) was the established cross-basis function. A 4th order polynomial function was used to specify the maximum number of lag days as 30, and xi was the heat index, dew-point temperature, daily maximum temperature and relative humidity respectively, which was used to illustrate the use of the natural spline function to control for long-term and seasonal trends. “Dow” and “holiday” were respectively week and holiday variables, used to remove confounding effects of week and holiday. The relative hazards were obtained and the lagged effects were visualized through the usage of the R language.

#### 2.2.2 Early warning modeling of heatstroke

Heatstroke occurrence has obvious time-series characteristics such as seasonality and is influenced by multiple factors (e.g., temperature, relative humidity, etc.) (22). Therefore, an attempt was made in this study to predict the number of heatstroke using two time series models, ARIMA and LSTM, along with several machine learning models such as regression decision tree, gradient boosting tree, SVR and random forest, from which the optimal algorithms were selected to be used as the main prediction tool for heatstroke early warning.

Autoregressive Integrated Moving Average (ARIMA), or Autoregressive Sliding Average Model, is a classical statistical method widely used for time series modeling and forecasting (23). Long short-term memory (LSTM) is a special variant of recurrent neural networks with a “gate” structure, which allows the network to converge better and faster, and can effectively improve prediction accuracy (24, 25).

Random forest is a powerful and flexible integrated learning algorithm commonly used for classification and regression problems (26). It is built on decision trees and improves the performance and generalization of the overall model by combining multiple decision trees (27). The algorithm uses Bootstrap sampling technique to randomly select multiple subsamples from the original dataset, each of which is used to train an independent decision tree (28). Its prediction results are based on the integration of multiple decision trees (Supplementary Figure 1). For the regression task the predicted value of the random forest is the average of all the decision trees. Suppose there are B decision trees and the predicted value of the ith tree is fi (*x*), then the predicted value of the random forest is:


$$\begin{array}{l} Ŷ\left(x\right)=\frac{1}{B}\overset{B}{\underset{i-1}{\sum }}f_{i}\left(X\right) \end{array}$$

The ARIMA and LSTM models were constructed by analyzing the time series of daily heatstroke occurrences, and both used rolling forecasts for better model predictions (29). The other machine learning models were trained with multiple features in mind and used static prediction in their forecasting. To facilitate the comparison of the models, each model used 2014–2018 data as the training set and 2019 data as the validation set. Parameter tuning of the models were performed by methods such as grid search to improve the generalization ability of the models.

## 3 Results

### 3.1 Descriptive statistics

This study analyzed the occurrence of heatstroke, related meteorological factors and comprehensive indicators from May to September in a southern city over a 6-year period from a variety of perspectives. The results showed that there were obvious seasonal fluctuations in the distribution of the number of heatstroke occurrences in this place, with the *sanfu* period being the high incidence time of heatstroke. The occurrence of heatstroke was mainly affected by the local temperature and relative humidity, and the calculated high-temperature heatwave and heat index had the strongest correlation with the number of heatstroke occurrences.

#### 3.1.1 Distribution of heatstroke occurrences

Descriptive statistical analysis of the number of heatstroke occurrences showed that July and August were the peak periods for heatstroke. From 2014 to 2019, the number of heatstroke occurrences in July was 1,753, accounting for 59.14 per cent of the total number; the number of heatstroke occurrences in August was 966, making up 32.59 per cent of the total number; and the number of heatstroke occurrences in June was 178, constituting 6.01 per cent of the total number; the number of heatstroke occurrences in May and September was comparable, with 24 and 43 occurrences respectively. The number of heatstroke occurrences from 2014 to 2019 showed a more pronounced seasonal variation, with a general trend of increasing and then decreasing (Supplementary Figure 2).

**Figure 1:**
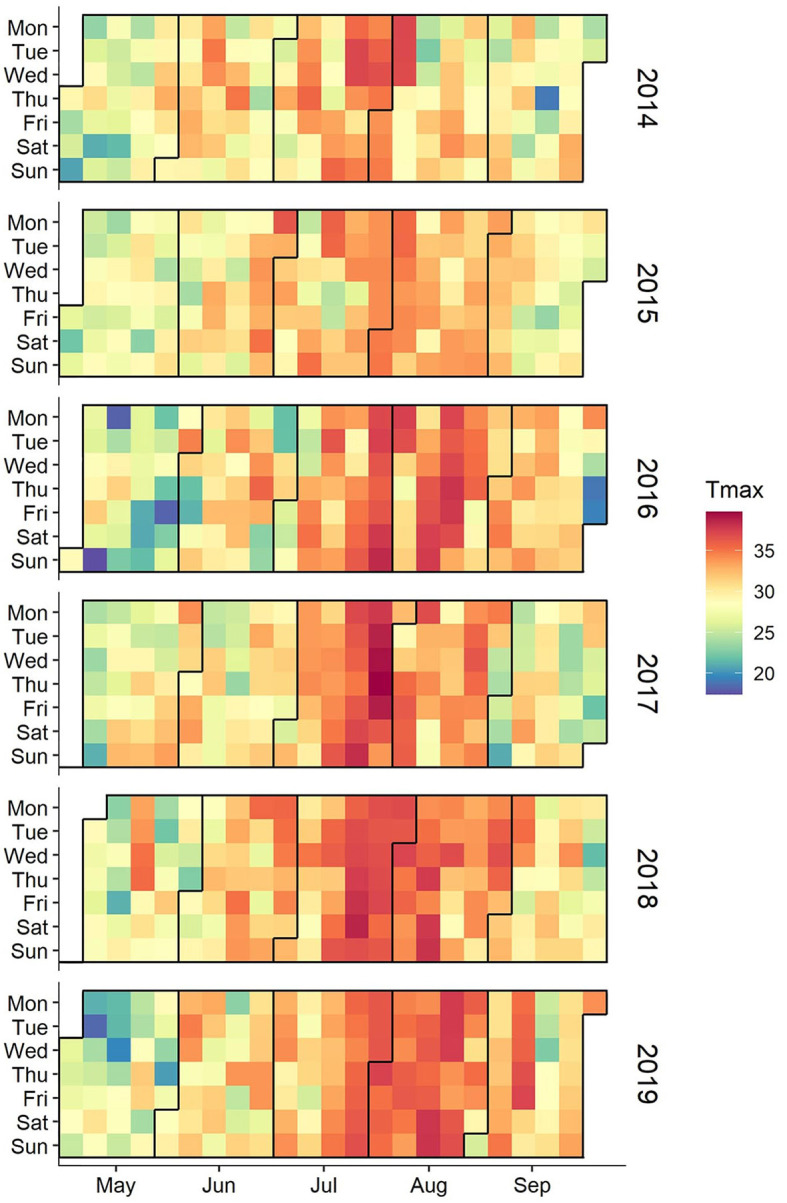
Date distribution of daily maximum temperature, May–September 2014–2019.

Through visual analyses of daily maximum temperatures, the daily distribution of daily maximum temperatures from May to September 2014–2019 was obtained, which is shown in Figure 1. Maximum temperatures concentrated in the *sanfu* period, and the *sanfu* days were the peak time for the occurrence of heatstroke. The highest number of heatstroke occurrences in *zhongfu* was 1,630, accounting for 54.99% of the total number. The number of heatstroke occurrences in the toufu, *mofu*, and non-*sanfu* days was relatively small, at 488, 227, and 619 respectively, constituting 16.46%, 7.66%, and 20.88% of the total number.

#### 3.1.2 Relevance analysis

**Figure 2:**
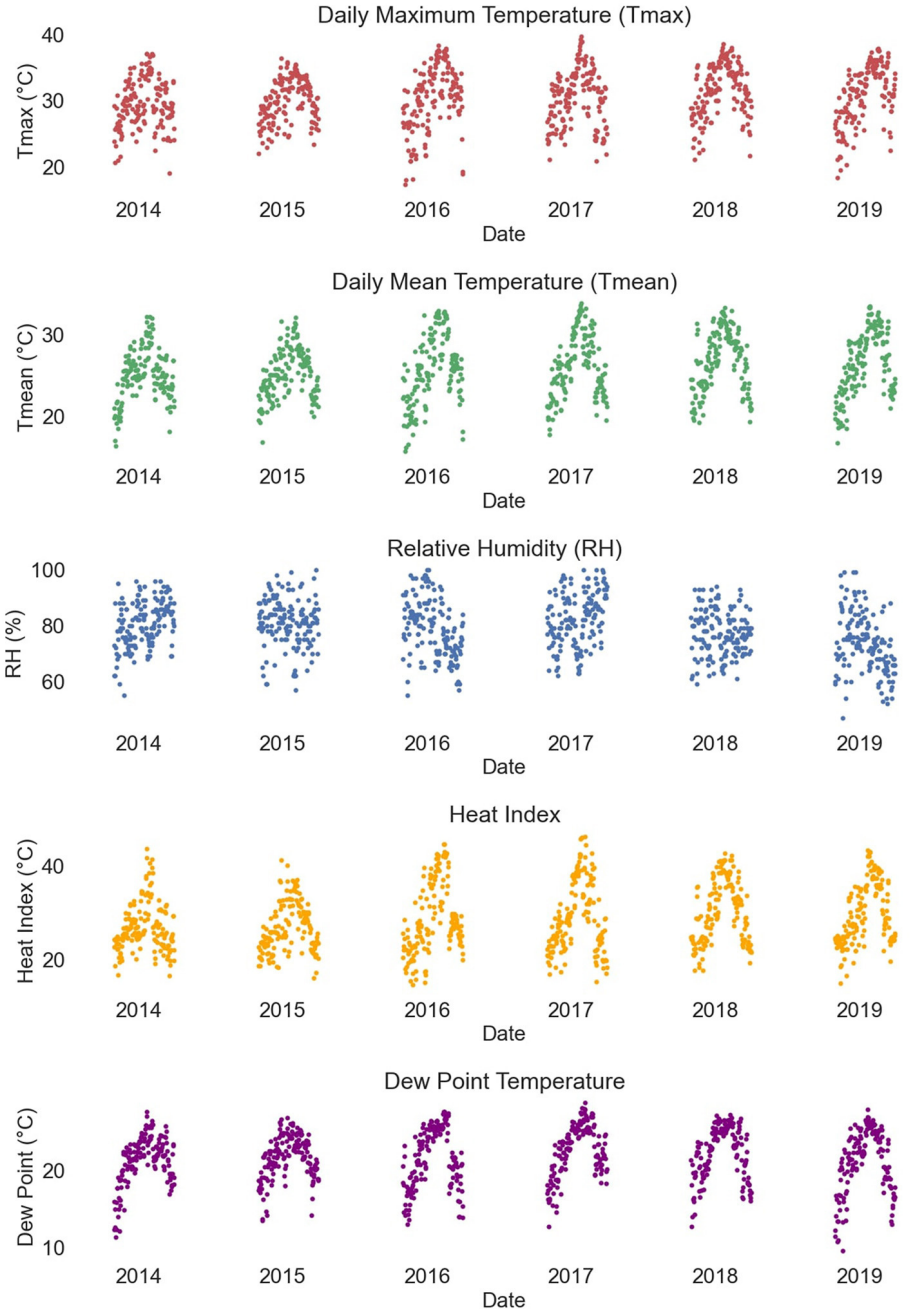
Time-series plot of meteorological data.

As shown in Figure 2, the daily maximum air temperature, daily mean air temperature, heat index, and dew-point temperature all exhibit distinct seasonal fluctuation patterns over the annual cycle. Further visualization analysis shows a significant positive correlation between temperature and the number of daily heatstroke events, and a clear temporal correspondence between the peak in the number of heatstroke events and the highest point in temperature (Supplementary Figure 3). Relative humidity shows some negative correlation with the number of daily heatstroke occurrences and corresponds to the peak in the number of daily heatstroke occurrences when the relative humidity drops to certain low points (Supplementary Figure 4).

**Figure 3:**
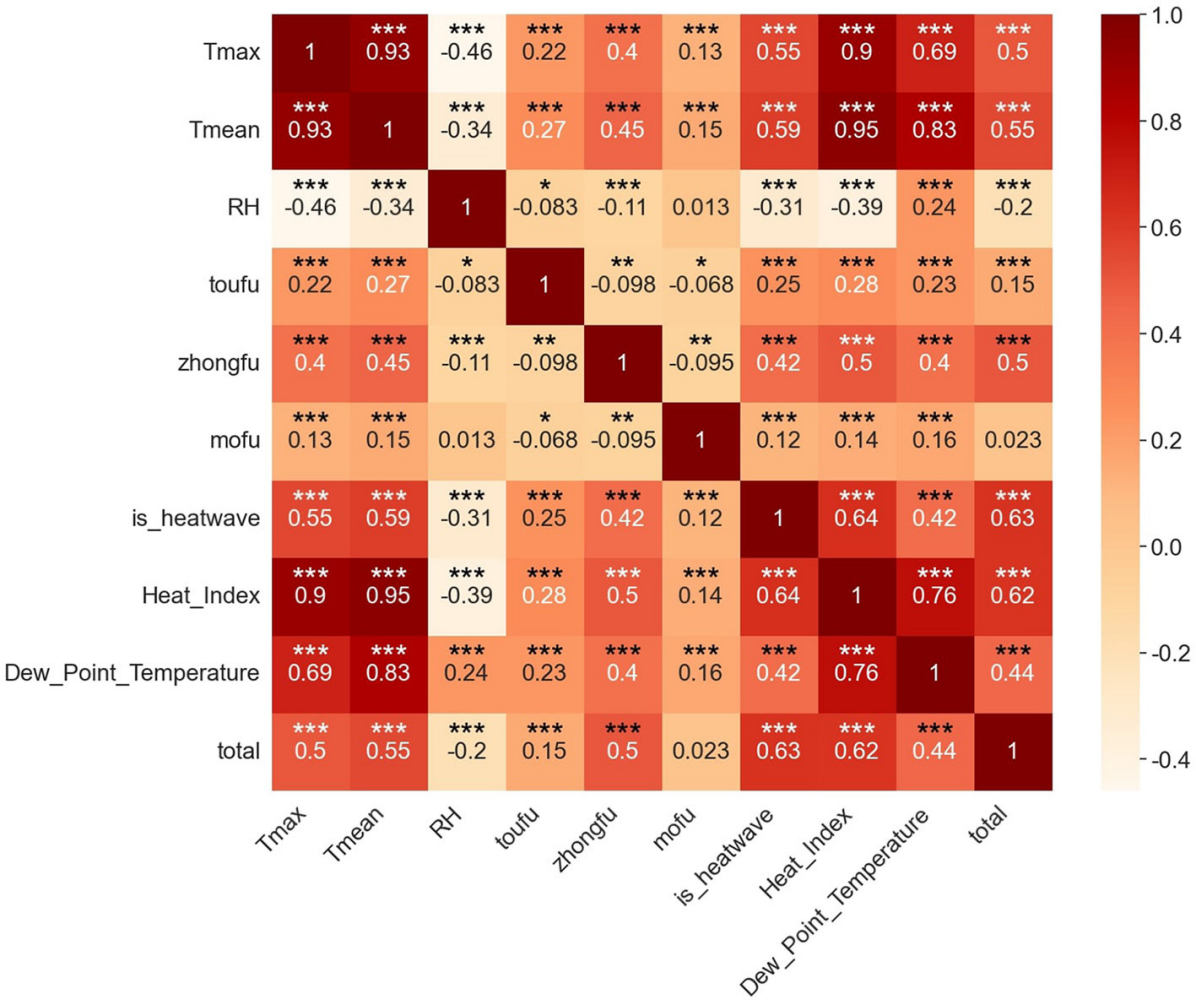
Heat map for correlation analysis. *represents statistical significance at the *p* < 0.05 level. **represents statistical significance at the *p* < 0.01 level. ***represents statistical significance at the *p* < 0.001 level.

The correlation of the variables in the data was visualized and the heat map obtained is shown in Figure 3, which reveals that the number of daily heatstroke occurrences shows a statistically significant correlation with daily maximum temperature, daily average temperature, relative humidity, *toufu, zhongfu*, high-temperature heatwaves, heat index, and dew-point temperature, with all *P*-values < 0.001, indicating a strong significance. The correlation coefficients between the number of daily heatstroke occurrences and the daily maximum temperature, daily average temperature are 0.5 and 0.55 respectively, indicating that the higher the temperature, the higher the likelihood of heatstroke occurrences. In addition, the correlation coefficient between the number of daily heatstroke occurrences and whether or not it is *zhongfu* is 0.5, indicating that the likelihood of heatstroke also increases during *zhongfu*. However, the correlation coefficient between the number of daily heatstroke occurrences and relative humidity is −0.2, indicating that the direct link between relative humidity and the number of heatstroke is not strong.

### 3.2 Cumulative and lagged effects

**Figure 4:**
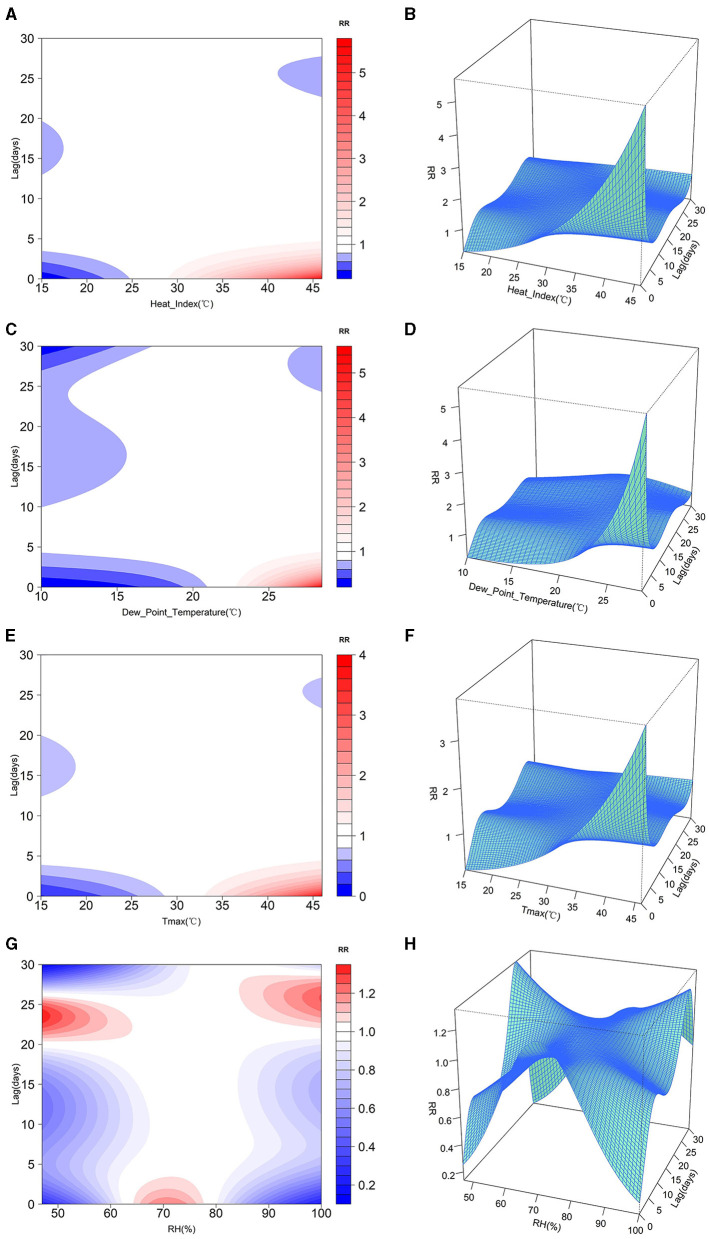
Relative risk of different meteorological factors and lagged days: **(A)** Heat Index 2D plot, **(B)** Heat Index 3D plot, **(C)** Dew-Point Temperature 2D plot, **(D)** Dew-Point Temperature 3D plot, **(E)** Daily Maximum Temperature 2D plot, **(F)** Daily Maximum Temperature 3D plot, **(G)** Relative Humidity 2D plot, **(H)** Relative humidity 3D plot.

Figure 4 presents the visualization of the results from the DLNM analysis, encompassing contour plots illustrating the changes in lag time, relative risk (RR), and meteorological data, along with three-dimensional representations depicting various meteorological factors, lag days, and RR values. Within the two-dimensional graphs, regions are color-coded, with red and blue areas signifying where RR is >1 and < 1, respectively. These graphical illustrations demonstrate the varying RR of heatstroke incidence in relation to shifts in heat index, dew-point temperature, maximum temperature, and relative humidity, indicating a non-linear association. The lagged effect of heat index, dew-point temperature and daily maximum air temperature on the number of heatstroke incidence was 0–5 days. When the daily heat index was >30°C, dew-point temperature was >23°C and maximum air temperature was >35°C, the risk of heatstroke increased significantly on the same day and within the following 5 days, and the risk of heatstroke decreased gradually with increasing lag time. Relative humidity had a lagged effect on the number of heatstroke occurrences and the effect varied with relative humidity. When the daily relative humidity was < 65%, the relative risk of the lagged 20–25 days was >1, and the risk of heatstroke increased; when the daily relative humidity was between 65 and 78%, the relative risk of the same day and the lagged 5 days was >1 and the risk of heatstroke was relatively high; when the daily relative humidity was > 85%, the relative risk of the lagged 22–28 days was >1, which shows that high humidity has a longer lagged effect on the number of heatstroke incidence.

### 3.3 Results and comparison of models for predicting the number of heatstroke

#### 3.3.1 Feature selection

In pursuit of enhancing the predictive accuracy of the model, this study initiated the process with the implementation of the Boruta Algorithm for feature selection. Boruta is a feature selection method grounded in random forests, which introduces randomized “shadow features” to compare against real features within an augmented feature matrix. The algorithm trains on this composite matrix and employs the importance scores of these shadow features as a reference baseline, thereby identifying a subset of real features that exhibit genuine relevance to the dependent variable. Given that the suggested depth for Boruta operates optimally with trees pruned to depths ranging from 3 to 7, our study configured each tree in the forest to a depth of 4, retaining default settings for all other parameters, including an estimator count set to “auto,” perc at 100%, alpha at 0.05, a two-step approach enabled (two_step = True), and a maximum iteration limit of 100. The resultant analysis identified day, daily maximum temperature (*T*_max_), daily mean temperature (*T*_mean_), relative humidity (RH), *zhongfu*, high-temperature heatwaves (is_heatwave), heat index (Heat_Index), and dew-point temperature (Dew_Point_Temperature) as variables exerting significant influence on the target variable.

#### 3.3.2 Model comparison

In order to compare the prediction ability of each model, three indicators, mean square error (MSE), root mean square error (RMSE) and coefficient of determination (*R*^2^), were selected for model evaluation in this study. The calculation formulas are as follows:


$$\begin{array}{r} MSE=\frac{1}{n}\overset{n}{\underset{i-1}{\sum }}|y_{i}-{ŷ}_{i}|\\ RMSE=\sqrt{\frac{1}{n}\overset{n}{\underset{i-1}{\sum }}{\left(y_{i}-{ŷ}_{i}\right)}^{2}}\\ R^{2}=1-\frac{\overset{n}{\underset{i=1}{\sum }}{\left(y_{i}-{ŷ}_{i}\right)}^{2}}{\overset{n}{\underset{i=1}{\sum }}{\left(y_{i}-ŷ\right)}^{2}} \end{array}$$

*n* is the number of samples; *y*_i_ is the *i*th observation; $\hat{y_{i}}$ is the *i*th predicted value; and ŷ is the mean of the observations.

**Figure 5:**
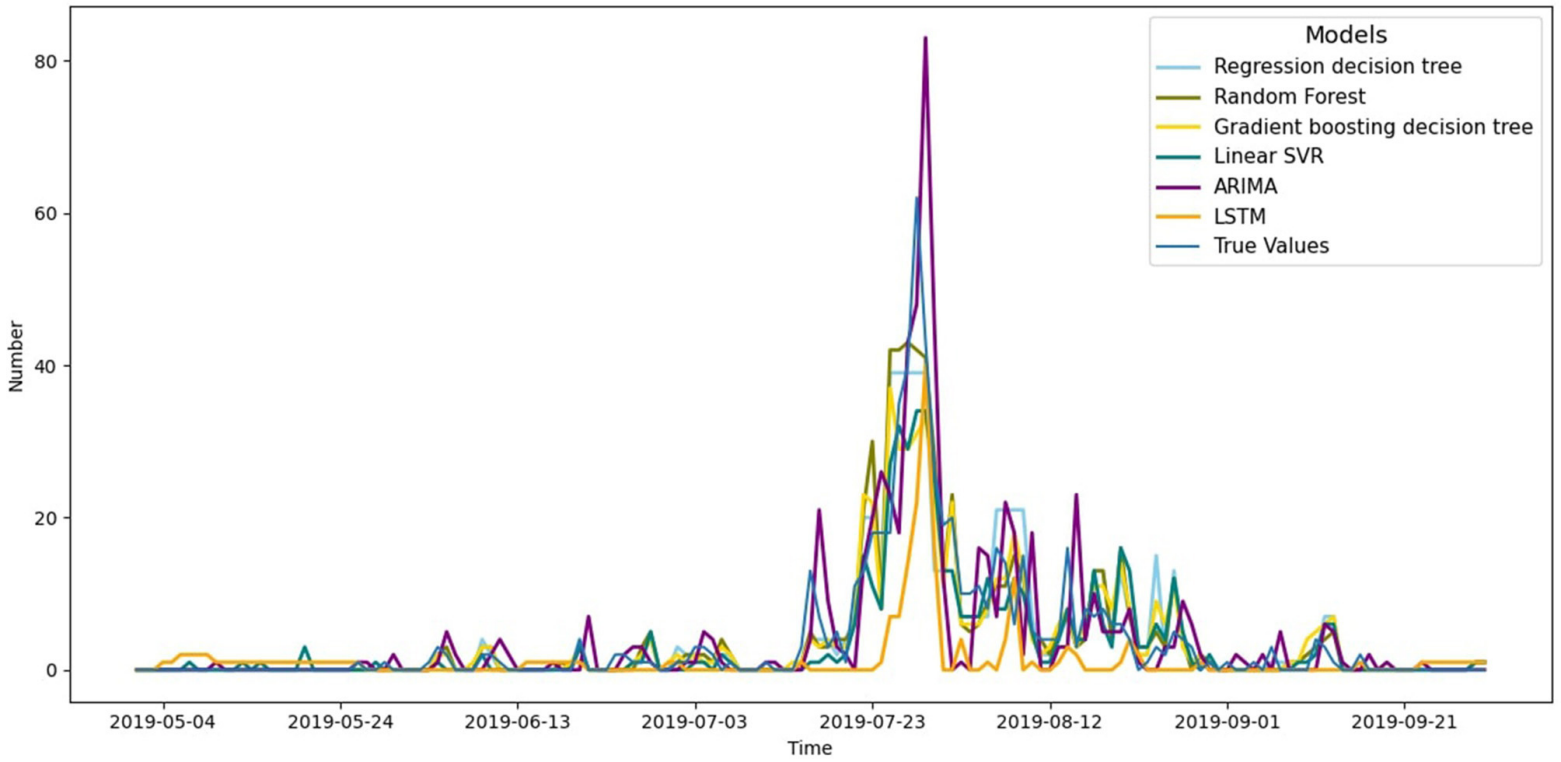
Forecasts of daily heatstroke numbers from May to September in 2019 by different model algorithms.

Residuals are defined as the difference between observations and model predictions. Mean square error (MSE) measures the extent to which the residuals are dispersed, while root mean square error (RMSE) measures the magnitude of residual fluctuations. RMSE is the same scale as MSE, but being on the same order of magnitude as the data points makes it easier to visually compare with the raw data (30). The lower the MSE and RMSE of the model, the higher the quality of the fit. The coefficient of determination *R*^2^ measures the strength of correlation between the predicted and actual values of the model, and its value tends to be closer to 1 indicates the stronger predictive ability the model has. The results of the evaluation of these indicators are shown in Table 1 and indicate that the random forest model stands out among all the compared models with its smallest MSE, RMSE, and *R*^2^ value closest to 1, which suggests that it has higher accuracy in predicting the number of heatstroke victims per day. In order to visualize the effectiveness of the different model algorithms in predicting the number of daily heatstroke occurrences from May to September 2019, a line graph of the actual number of observations against the predictions of the four models was plotted, using time as the horizontal coordinate and the number of daily heatstroke occurrences as the vertical coordinate, as shown in Figure 5.

**Table 1:** Comparison of evaluation indicators for different models.

**Model name**	**MSE**	**RMSE**	***R* ^2^**
Regression decision tree	15.13	3.89	0.79
Random forest	12.74	3.57	0.82
Gradient boosting decision tree	14.64	3.82	0.80
Linear SVR	14.86	3.85	0.80
ARIMA	34.58	5.89	0.52
LSTM	44.86	6.70	0.38

#### 3.3.3 Random forest model

In this study, the random forest model was hyper-parametrically optimized by a grid search method to determine the optimal parameter configuration: the maximum depth of the decision tree was set to 4; the minimum number of samples to be included in each leaf node was set to 1; and the minimum number of samples required to split a node was 4; the model as a whole consisted of 13 decision trees.

**Figure 6:**
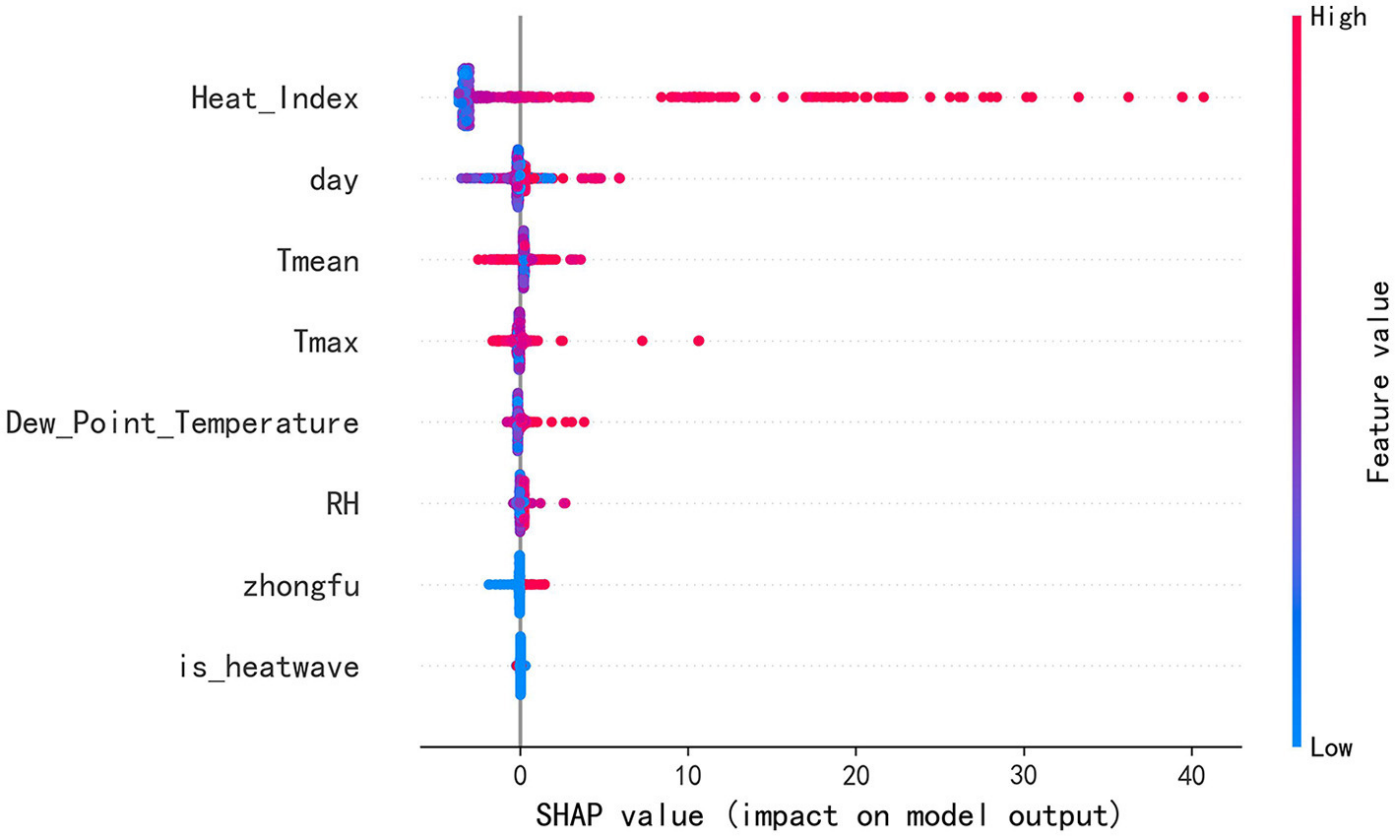
SHAP summary plot.

Furthermore, this study calculated the SHAP values and SHAP interaction values for the finalized model and presented them visually, and the results obtained are exhibited in Figure 6. The results show that the thermal index received the highest feature importance score and played a key role in the model prediction process. This finding is consistent with thermodynamic principles and established medical a priori knowledge, the latter suggesting that the onset of heatstroke is closely related to high temperature and relative humidity. Daily mean temperature, daily maximum temperature, relative humidity, and dew-point temperature also exhibited notable significance in the model. The feature importance of the date in the time series was notably high, suggesting a potential periodicity in heatstroke cases throughout the months. Although the feature importance score for the *zhongfu* period was relatively low, the graphical depiction clearly illustrated a pronounced positive impact of *zhongfu* on the escalation of heatstroke cases, aligning with the descriptive statistical findings of this research. By comparison, the feature related to high-temperature heatwaves had a lesser role in the model, yet it still contributed to the model's performance to some extent.

In order to assess the robustness of the constructed random forest regression model under different conditions, this study conducted a sensitivity analysis by adjusting the values of each feature individually and monitoring the possible effects of these adjustments on the model performance. By comparing the differences in model scores before and after adjusting the feature values, we assessed the specific impact of different features on model performance (Supplementary Figure 5). The results show that changes in the values of features such as heat index, daily mean temperature, daily maximum temperature, onset day, and dew-point temperature had some impact on the model, but the impacts were all small, indicating that the random forest regression model developed in this study has good stability and robustness.

## 4 Discussion

In the context of rising global temperatures, the onset of high-temperature red alerts is occurring earlier, their durations are extending, the affected areas are broadening, their intensities are amplifying, and their extremities are enhancing. Consequently, the incidence of heatstroke, a meteorologically sensitive illness, is anticipated to rise. Therefore, the prediction and early warning of heatstroke are vital, which can enable the relevant departments and the public to get the relevant information in time, which is conducive to the adoption of protective measures in advance, to avoid health risk, and to reduce the damage to health. This study analyzed the cumulative and lagged effects of these factors on heatstroke by screening the influential features of heatstroke and constructing DLNM model analysis. Moreover, various machine learning methods were tried to construct a prediction model for the number of heatstroke victims, and after comparison, we found that the random forest model had the best prediction effect. Through the sensitivity analysis, the model showed high robustness, which indicated that the model would still be able to maintain a highly reliable prediction performance even in the face of some parameter variations or uncertainties.

### 4.1 Influence of meteorological factors on the number of heatstroke victims

Meteorological factors have a significant impact on summer heatstroke. This study has found that there existed a high correlation between the number of heatstroke cases and the following meteorological variables: high-temperature heatwaves, heat index, daily mean temperature, and daily maximum temperature, as evidenced by substantial correlation coefficient values, implying that they not only directly lead to discomfort, but also may cause high-risk health problems, especially in areas where extreme heat is infrequent (31). However, the feature importance of high-temperature heatwaves was not prominent in the random forest predictive model. Current research on the daytime, nighttime, and compound heatwaves suggests that nighttime heatwaves predominantly occur in low-latitude regions and are typically accompanied by high humidity conditions during nighttime, and that nighttime warmth may impose additional health risks (32). Furthermore, in recent years, heatwaves have exhibited trends of longer durations, greater spatial extents, and slower movement, with slow-moving heatwaves indicative of prolonged high temperatures, potentially having a substantial impact on heatstroke incidences (33). Consequently, future studies could incorporate a broader range of data related to heatwaves to enhance the precision of predictive early warning systems.

Regarding time series aspects, the total number of heatstroke cases during the *sanfu* periods accounted for 79.11% of the total heatstroke occurrences, closely aligning with the peak timing of heatstroke incidents. Particularly during the *zhongfu* phase, temperatures typically reached seasonal highs, and this temporal characteristic exhibited a significant correlation with the heatstroke incidence, reaching 0.5, further confirming high temperatures as a pivotal meteorological factor in heatstroke occurrences. Within the studied region, the effect of relative humidit on heatstroke was relatively minor. As the DLNM model analysis suggests, the impact of relative humidity on heatstroke morbidity was neither linear nor monotonous but an inverted-U shape. Relative humidity displayd a pronounced short-term lagged effect within the range of 65%−78%, while showing more evident long-term lagged effects when relative humidity was below 65% or above 85%, leading to a lower Pearson correlation coefficient. This inverted-U pattern might result from dehydration under low humidity in high temperatures and severe hindrance of heat dissipation under high humidity, both of which disrupt thermoregulation and elevate heatstroke risk over extended periods (34).

Moreover, the DLNM model analysis reveals a lagged effect of heat index and dew-point temperature on heatstroke incidence, with a marked increase in risk on the day of exposure and up to following 5 days once certain threshold values are surpassed (35). High-temperature heatwaves, heat index, daily mean temperatures, and daily maximum temperatures all exert noticeable lagged effects on heatstroke occurrences. In preventing and managing heatstroke, the delayed impacts of these meteorological factors must be fully considered, and appropriate protective measures should be implemented to mitigate heatstroke incidents.

### 4.2 Forecasting and early warning models

The frequency and duration of extreme temperature events are increasing (31), and the number of heatstroke victims is likely to show a continuous increase in the future (11). It is necessary to construct a forecasting and early warning model for the prediction of the number of heatstroke victims to provide early warning of heatstroke incidence (36). Based on the analysis results of DLNM, this study experimented with a variety of machine learning algorithms to construct a forecasting and warning model for heatstroke, and chose to adopt the random forest model, which is the most effective and robust model, to predict the number of people suffering from heatstroke per day by using multiple meteorological factors and time factors.

Comparing the structure of the decision trees within the final models, commonalities in the branching structure are visible at certain levels, which maps to a consistent understanding of the key predictors in the model. For example, the heat index and average daily temperature are prominent in most trees, implying that these two features contribute more to predicting the target variable in the overall model. At the same time, individual decision trees were observed to pay more attention to additional factors such as “day,” “*zhongfu*,” and “high-temperature heatwaves,” signaling that the model has a certain degree of versatility and is able to make more adaptable predictions for different data patterns.

Synthesizing the analysis of the DLNM model and the results of the comparison of the prediction models, this study proposes a comprehensive early warning mechanism for heatstroke. The core strategy is to use the random forest model to make accurate heatstroke number predictions. Based on the predicted data and the set warning thresholds, an early warning is implemented when the model predicts a high risk of heatstroke events, and further calculation of the relative humidity, heat index, and dew-point temperature is made. If one of them is identified as a driver of heatstroke occurrences beyond the warning thresholds, then a reinforced warning process for at least five consecutive days is started. At the same time, if the relative humidity is < 65 per cent on that day, another warning is issued on the 20th to 25th day after that day, and if it is >85 per cent, another warning is issued on the 22nd to 28th day after that day.

The model could provide real-time early warning information to governments, medical institutions and other public health departments, and promote the development of appropriate preventive measures. By using the model, public health departments can intervene early to improve public health and safety emergency response capabilities, enhance group health protection, and reduce the incidence of heatstroke.

### 4.3 Research programmes and prospects

The scope of this paper is mainly limited to a specific region in southern China, and thus it is difficult to directly apply the conclusions obtained so far to cities in other geographical environments or climatic conditions. In terms of the selection of meteorological factors, this study covers a relatively limited number of indicators and does not take into account factors such as wind speed, air pressure and weather phenomena. Future research endeavors will broaden the scope of data collection to encompass climatic data from diverse regions, facilitating inter-regional comparative analyses. This enhanced dataset will incorporate a wider array of meteorological variables, including wind velocity, atmospheric pressure, nocturnal heatwaves, compound heatwaves, and the velocity of heatwave movement, aligning with the forefront of meteorological research domains. Such comprehensive data integration aims to enhance the precision of predictive early warning systems.

Exertional heatstroke poses a persistent threat to individuals exposed to high temperatures, with young, healthy individuals of higher body mass index exhibiting an elevated risk, as evidenced in recent literature (37). The diagnostic criteria for occupational heatstroke released by the Chinese Center for Disease Control and Prevention in 2019 highlighted outdoor occupations such as construction, engineering, agricultural labor, and sanitation work as prevalent causes during summer months. Furthermore, intense activities during summer, including sports competitions and military drills, significantly contribute to heatstroke incidents. Subsequent studies could, therefore, stratify participants based on occupation and duration of outdoor exposure, in addition to gender and age, to create detailed population profiles. Integrating these profiles with the city-specific meteorological factors would enable a holistic analysis and the targeted delivery of heatstroke warnings.

The aspiration is that these methodologies will augment the universality and accuracy of heatstroke alerts, thereby furnishing more scientifically grounded approaches for the prevention and control of urban heatstroke diseases.

## 5 Conclusion

In this study, a distributed lag nonlinear model was used to investigate the lagged and cumulative effects of various climatic factors on heatstroke, and a forecasting model for daily heatstroke occurrences was constructed using the random forest algorithm. The early warning strategy for heatstroke shows that exposure to hot and humid weather tends to increase the risk of heatstroke, and their effects are not limited to the day, but can last for days afterwards. These findings may inform government departments, medical institutions and other organizations of more accurate early warning of heatstroke risks, thus improving public health and safety emergency response capabilities, and reducing the damage to health caused by hot weather.

## Data availability statement

Publicly available datasets were analyzed in this study. This data can be found at: http://www.ncmi.cn.

## Author contributions

HX: Conceptualization, Data curation, Funding acquisition, Methodology, Project administration, Resources, Validation, Visualization, Writing – original draft, Writing – review & editing, Formal analysis, Investigation, Supervision. SG: Data curation, Formal analysis, Methodology, Resources, Visualization, Writing – original draft, Writing – review & editing. XS: Data curation, Formal analysis, Methodology, Resources, Visualization, Writing – original draft, Writing – review & editing. YW: Investigation, Methodology, Resources, Visualization, Writing – original draft, Writing – review & editing. JP: Data curation, Methodology, Resources, Visualization, Writing – original draft, Writing – review & editing. HG: Writing – original draft, Writing – review & editing. YT: Conceptualization, Funding acquisition, Resources, Software, Supervision, Validation, Writing – original draft, Writing – review & editing. AH: Conceptualization, Funding acquisition, Resources, Software, Supervision, Validation, Writing – original draft, Writing – review & editing.
