# Supplementary material for: Machine learning-based analysis and prediction of meteorological factors and urban heatstroke diseases
Source: Front Public Health. 2024 Jul 22;12:1420608. doi: 10.3389/fpubh.2024.1420608 (PMC11299116; doi:10.3389/fpubh.2024.1420608)
Supplement: Supplementary file 1 [file Table_1.DOCX]

Supplementary Material

Machine Learning-based Analysis and Prediction of Meteorological Factors and Urban Heatstroke Diseases

Hui Xu^1^, Shufang Guo^1†^, Xiaojun Shi^1†^, Yanzhen Wu^1^, Junyi Pan^1^, Han Gao^2^, Yan Tang^1*^, Aiqing Han^1*^

^1^ School of Management, Beijing University of Chinese Medicine, Beijing, China

^2^ School of Humanities, Beijing University of Chinese Medicine, Beijing, China

*** Correspondence:**

Yan Tang

tangyan97_1017@sina.com

Aiqing Han

aqhan@hotmail.com

# Supplementary Figures and Tables

## Supplementary Tables

**Supplementary Table 1.** *Sanfu* periods from 2014 to 2019.

| **Year** | ***Toufu*** | ***Zhongfu*** | ***Mofu*** |
| --- | --- | --- | --- |
| 2014 | July 18 - July 27 | July 28 - August 06 | August 07 - August 16 |
| 2015 | July 13 - July 22 | July 23 - August 11 | August 12 - August 21 |
| 2016 | July 17 - July 26 | July 27 - August 15 | August 16 - August 25 |
| 2017 | July 12 - July 21 | July 22 - August 10 | August 11 - August 20 |
| 2018 | July 17 - July 26 | July 27 - August 15 | August 16 - August 25 |
| 2019 | July 12 - July 21 | July 22 - August 10 | August 11 - August 20 |
