# Supplementary material for: Machine learning-based analysis and prediction of meteorological factors and urban heatstroke diseases
Source: Front Public Health. 2024 Jul 22;12:1420608. doi: 10.3389/fpubh.2024.1420608 (PMC11299116; doi:10.3389/fpubh.2024.1420608)
Supplement: Supplementary file 2 [file Table_2.DOCX]

Supplementary Material

Machine Learning-based Analysis and Prediction of Meteorological Factors and Urban Heatstroke Diseases

Hui Xu^1^, Shufang Guo^1†^, Xiaojun Shi^1†^, Yanzhen Wu^1^, Junyi Pan^1^, Han Gao^2^, Yan Tang^1*^, Aiqing Han^1*^

^1^ School of Management, Beijing University of Chinese Medicine, Beijing, China

^2^ School of Humanities, Beijing University of Chinese Medicine, Beijing, China

*** Correspondence:**

Yan Tang

tangyan97_1017@sina.com

Aiqing Han

aqhan@hotmail.com

# Supplementary Figures and Tables

## Supplementary Tables

**Supplementary Table 2.** Variables and their meanings.

| **Variable** | **Meaning** | **Description** |
| --- | --- | --- |
| Date | Date of onset | e.g. 2014/5/1 |
| Dow | Day of the week | e.g. Monday |
| Holiday | Holiday | 1 for yes, 0 for no |
| Year | Year | e.g. 2014 |
| Month | Month | e.g. March |
| Day | Day | e.g. 1^st^ |
| Tmean | Average daily temperature | e.g. 21 (℃) |
| Tmax | Daily maximum temperature | e.g. 29.3 (℃) |
| RH | Relative humidity | e.g. 73 (%RH) |
| Total | Number of heatstrokes per day | e.g. 0 (persons) |
| Pop | Total population | e.g. 7660504 (persons) |
| heatwave | heatwave | 1 for yes, 0 for no |
| *toufu* | The beginning part of *sanfu* | 1 for yes, 0 for no |
| *zhongfu* | The middle part of *sanfu* | 1 for yes, 0 for no |
| *mofu* | The ending part of *sanfu* | 1 for yes, 0 for no |
| Heat index | Heat index | e.g. 22.7 (℃) |
| Dew-point temperature | Dew-point temperature | e.g. 15.9 (℃) |
