# Supplementary figures and images for: Machine learning-based analysis and prediction of meteorological factors and urban heatstroke diseases
Source: Front Public Health. 2024 Jul 22;12:1420608. doi: 10.3389/fpubh.2024.1420608 (PMC11299116; doi:10.3389/fpubh.2024.1420608)

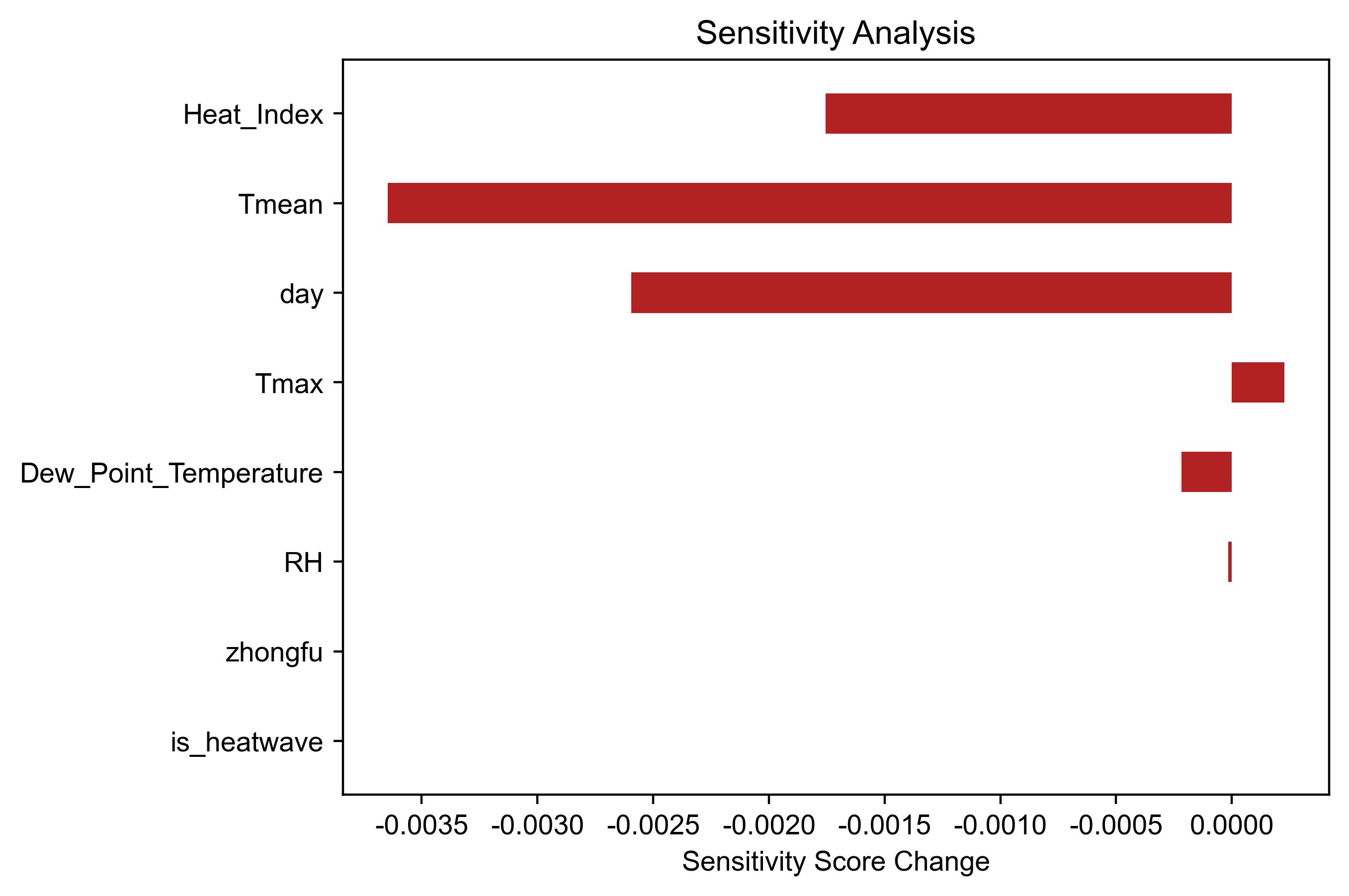

Supplement: Supplementary file 7 [file Image_5.JPEG]
